# Diseases of the Heart and Circulation

**Published:** 1904-01-02

**Authors:** 


### DISEASES OF THE HEART AND CIRCULATION.

(*Concluded from page* 227.)

**Rise of Blood-pressure in Later Life.**—A discussion on this important subject, during the early part of this year, was initiated by lectures of Allbutt,[2] who said that arterial disease was not necessarily attended by a rise of blood pressure. The more senile forms of arterial disease may be divided into those in which the pressure was raised and those in which it was normal or even low. The first kind tended to death from apoplexy, the latter to obliterative changes in the cerebral arteries with softening. The rise in pressure seen in elderly persons, especially in those who had led a sedentary life, with positive or relative excess in food, is probably due to increase in the viscosity of the blood. Arterio-sclerosis is the result, rather than the cause, of this increase of pressure. With Hill and Barnard's sphygmometer the blood-pressure of adults varies between 95 and 120 mm. Hg. In Bright's disease it may rise to 250 mm. Hg. Between disease of the arterial tree and blood-pressure there is no direct relation. A persistent fall of pressure in a case in which it has hitherto been high usually signifies heart failure. When the subject of arterio-sclerosis begins to be short of breath he has entered upon the last stage of his pilgrimage. In elderly persons, in whom lead, syphilis, alcohol, and kidney disease can be excluded, arterio sclerosis can be divided into two kinds, one the result of persistent high pressure, the other the result of some more intimate causes, perhaps some unknown poison or the tooth of time. When calcification is a prominent and early feature in the superficial vessels pressures are apt to be moderate or even low. In those cases in which the pressure is high it is of great importance to detect the disease at an early stage. Suspicion from the feel of the pulse can be verified by the use of a sphygmometer. Treatment is difficult, for though saline purgatives, restriction of foods, and exercise may reduce the blood-pressure, yet this will often bring about listlessness and malaise, due to the reduction of pressure below the acquired habit, and so the patient be dissatisfied. In many men badly affected with recurrent gout, if free from kidney disease and of temperate habits, the arterial pressure does not rise. "Suppressed gout" is a label for a heterogeneous bundle of maladies, but a great many of these are, in reality, cases of this arterial plethora. The cause of the rise in blood-pressure is viscosity of the blood, but the origin of this is obscure; possibly it is due to products of faulty metabolism. Broadbent[3] said that the rise in blood-pressure was due to peripheral obstruction, which arose either in the arterioles or in the capillaries, and probably in the latter, as suggested by the miliary aneurysms in the minute arterioles. Allbutt's use of the term viscosity is misleading, for the cause of the obstruction in the capillaries may not be viscosity in the ordinary physical sense. The capillaries are extremely sensitive to the presence of substances in the blood, and minute traces of bodies due to imperfect metabolism may increase capillary obstruction. Arteriole obstruction under nervous stimulation can raise the blood pressure so as to give rise to increased secretion of urine, but it is not the cause of the persistent high pressure of arterio-sclerosis. Russell[4] said that certain conditions of blood—sufficiently indicated by the word "impurity"—lead to a tightening-up and thickening of vessel-walls, which is often mistaken for true structural alteration. The recurrence of this hypertonus leads ultimately to structural changes in the vessels, and along with these changes there may or may not be raised blood pressure. Allbutt's use of the term "viscosity" suggests some physical alteration in the blood which by impeding its flow leads to heightened pressure, but to no active contractile reaction on the part of the vessel wall. But clinical observation shows active contraction of blood-vessels. In reply to these criticisms, Allbutt[5] says that it is unknown whether blood into which, let us say, purin bodies have been introduced, wets membranes less than normal blood as Broadbent suggests, or whether it is of higher viscosity as he suggests. The question is one for future experiment. In reply to Russell, Allbutt denies that arterial spasm comes first and raises the mean arterial pressure. Campbell[6] adds to the discussion an interesting paper. In physiological conditions the resistance in the capillaries is so low as to be negligible, the chief vascular resistance is in the arterioles. Physiological modifications of vascular resistance are almost wholly due to active alterations in the arterioles, modifications in the frictionality (vital or mechanical) of the blood playing but an infinitesimally small part. The degree of systemic arterial blood pressure is essentially dependent on the degree of arteriolar resistance. In disease it is probably the same mechanism at work. A toxæmia causes contraction of the systemic arterioles, except those within the kidneys, so that the elimination of the poison is favoured. And Russell correspondingly found that in hypertonus the muscular coat was hypertrophied save in the kidneys, where it tends to be atrophied. The objection to the view that the increase in resistance takes place in the capillaries is that whereas, if it takes place in the arterioles, a fractional increase is sufficient to account for the facts; in the capillaries the normal resistance would have to be increased twenty-fold. Morison[7] is of opinion that if muscular hypertrophy is due primarily to sustained hypertonus, it is soon increased by having to deal with the more persistent inelasticity of connective tissue hyperplasia, which is an active cause of difficulty in the circulation.

[2] Lancet, Jan. 17 and March 7, 1903. [3] Lancet, Feb. 7, 1903. [4] Ibid. [5] Lancet, Feb. 14, 1903. [6] Ibid. [7] Lancet, Feb. 23, 1903.

